# Management and Treatment of External Hemorrhoidal Thrombosis

**DOI:** 10.3389/fsurg.2022.898850

**Published:** 2022-05-03

**Authors:** Arcangelo Picciariello, Marcella Rinaldi, Ugo Grossi, Luigi Verre, Michele De Fazio, Agnese Dezi, Giovanni Tomasicchio, Donato F Altomare, Gaetano Gallo

**Affiliations:** ^1^ Department of Emergency and Organ Transplantation and Inter-Department Research Center for Pelvic Floor Diseases (CIRPAP), University Aldo Moro of Bari, Bari, Italy; ^2^ II Surgery Unit, Regional Hospital Treviso, DISCOG, University of Padua, Treviso, Italy; ^3^ Department of Medicine, Surgery and Neurosciences, Unit of General Surgery and Surgical Oncology, University of Siena, Siena, Italy

**Keywords:** hemorrhoidal disease, external hemorrhoidal thrombosis, hemorrhoidectomy, surgery, pregnancy

## Abstract

**Background:**

External hemorrhoidal thrombosis (EHT) is a common complication of hemorrhoidal disease. This condition causes extreme pain, likely resulting from internal anal sphincter hypertonicity, which traps the hemorrhoids below the dentate line thus leading to congestion and swelling. The choice of treatment remains controversial and both conservative and surgical options have been proposed in the last decades.

**Methods:**

This mini-review focuses on the most relevant studies found in literature evaluating conservative and surgical management of EHT. Special conditions such as pregnancy and EHT in elderly patients have been considered.

**Results:**

Traditionally, symptoms duration represents the discriminant in the choice between medical and surgical treatment. Several Coloproctological Societies considered conservative treatment as the first-line approach to EHT and a variety of options have been proposed: wait and see, mixture of flavonoids, mix of lidocaine and nifedipine, botulinum toxin injection and topical application of 0.2% glyceryl trinitrate. Meanwhile, different surgical treatments are recommended when EHT fails to respond to conservative management or when symptoms onset falls within the last 48–72 h: drainage with radial incision, conventional excision, excision under local anesthesia and stapled technique.

**Conclusion:**

The management and treatment of EHT is still controversial since no specific guidelines have been published. Both medical and surgical treatment have been proven effective but randomized clinical trials and structured consensus-based guidelines are warranted.

## Introduction

Hemorrhoidal thrombosis is one of the most frequently diagnosed complication of hemorrhoidal disease that can involve both the internal and the external hemorrhoidal plexus (1).

External hemorrhoidal thrombosis (EHT) commonly occurs in young adults of both sexes (2), representing distended vascular tissue in the anal canal, distal to the dentate line, covered by richly innervated anoderm (3). This condition causes extreme pain, likely resulting from internal anal sphincter hypertonicity, which traps the hemorrhoids below the dentate line thus leading to congestion and swelling (2, 3).

Etiology of EHT is still a matter of debate. It is linked to an increased intravenous pressure in the hemorrhoidal plexus which leads to rupturing of the endothelial lining initiating thrombosis (4, 5). Young age, hard stool, constipation, excessive physical effort and use of dry toilet paper combined with wet cleaning methods after defecation appear to promote EHT development, whereas the use of bathtub or shower before sleep seem to have a protective role (4, 5). Pregnancy, commonly considered a risk factor for EHT, has no significant relationship, except for childbirth (5). Occasionally, recurrent EHT could be associated with administration of L-asparaginase, in patients with Philadelphia chromosome-positive acute lymphoblastic leukemia (6).

There is neither a classification nor a consensus agreement for evaluating the presence and severity of EHT. For this reason, the choice of the type of treatment remains controversial (7).

The decision-making process usually depends on timing since symptom onset, with surgical treatment being favored with symptoms onset occurring in the preceding 72 h (8, 9). Severity of symptoms and patient preference should also be considered (10). Conservative treatment is mainly symptomatic, with use of analgesics, nifedipine or glyceryl trinitrate (GTN), activity reduction and laxatives (11). Surgical treatment options include incision and evacuation of the thrombus (12) or excision of the thrombosed hemorrhoid (10).

## Methods

Published literature was searched using PubMed to identify publications reporting the treatment and the clinical assessment of EHT between January 1, 2000 and January 1, 2022.

Key words for the search were: “external hemorrhoidal thrombosis”, “thrombosed hemorrhoids”, “acute thrombosed hemorrhoids”, “external thrombosis hemorrhoids”, and “acute hemorrhoids”.

Screening of articles was performed at the abstract level by four authors (AP, AD, GT, and MR), excluding studies not meeting eligibility criteria (i.e., medical and surgical treatments, outcomes in patients affected by EHT, EHT during pregnancy and in elderly patients) where these could be readily determined from the abstract alone.

Study characteristics and outcome data were extracted independently onto a Microsoft Excel spreadsheet. The following data were extracted for each study: first author, year of publication, authors’ country, study design and length (in years), number of patients, patients’ demographics (gender, age), type and duration of symptoms, etiology of EHT, type of medical or surgical treatment, recurrence rate.

## Clinical Assesment

**Figure 1:**
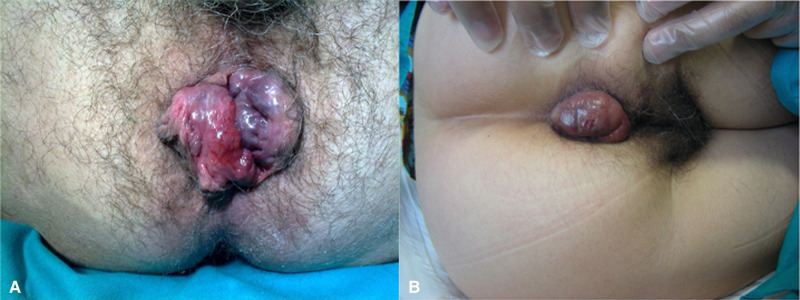
**(A**,**B**) External thrombosed hemorrhoids and anal lump.

The diagnosis of EHT is clinical, based on accurate collection of anamnestic data and on proctological examination. When collecting anamnestic data, patients usually refer an episode of straining with constipation, physical effort or diarrhea (3). Classic symptoms of this condition are acute and invalidating anal pain with appearance of a perianal lump (11, 13). Patients with EHT complain of sudden onset of anal pain with appearance of a visible, bluish perianal lump and a certain degree of internal anal sphincter hypertonia. Bleeding is infrequent and occurs only when the thrombus leads to ulceration of the underlying skin (14) (**Figure 1A,B**). Pain associated with EHT can be extremely intense and debilitating for the patient, requiring immediate management. If left untreated, symptoms may take several days or weeks to resolve (2, 15). The anorectal examination is fundamental for correct diagnosis and is typically performed with the patient lying in left lateral decubitus position. It consists of visual inspection, digital examination and anoscopy. Inspection, in case of EHT, allows identification of a bluish perianal lump, which must be differentiated from complicated internal hemorrhoids and pigmented anal melanoma. EHT are covered by anoderm, whereas complicated internal hemorrhoids are covered by anal mucosa. Pigmented anal melanoma can be excluded in case of sudden appearance of the bluish perianal discoloration (11). Digital rectal examination allows evaluation of the resting sphincter tone, which is usually increased in patients with acute hemorrhoidal crisis (16).

## Conservative Management and Outcomes

Several Coloproctological Societies considered conservative treatment as the first-line approach to EHT (16, 17).

Traditionally, the most important discriminant in the choice between medical or surgical treatment is symptoms duration when reaching medical attention. Medical approach is reserved to patients experiencing symptoms beyond 48–72 h from onset (3, 11, 12, 18). However, there is no evidence in literature that conservative management is best used in cases of early onset of symptoms. It seems to be based on clinical experience handed down over the years. Sammarco et al. and Chan et al. recommended to base the choice of approach not only on timing since onset of symptoms but also on the severity of patient’s symptoms and needs (10, 14).

A variety of conservative treatment options have been proposed (16). The first level of treatment is “wait and see” and includes a combination of local hygiene measures, ointments, sitz baths, high-fiber diets, increased oral intake of fluid, stool softeners, oral and topical analgesics (15–17). Gebbensleben et al. (19) proposed a strict management policy: no water, showering, washcloth use, wet wipes, soap or shower gel for hygiene after defecation, but only use of a smooth dry sheet of toilet paper for one to two weeks. Patients were asked to complete a questionnaire at study entry and six months later. At follow-up, 62.5% described themselves as “healed” or “ameliorated”, a recurrence was suspected in 21.3%, and 45.8% reported persistence of at least one symptom (i.e., itching, pain, sore anus, bleeding and burning).

A recent randomized, controlled, triple blind trial demonstrated the efficacy of oral intake of a mixture of diosmin, troxerutin and hesperidin in the treatment of acute hemorrhoid crisis (20). The mixture of flavonoids showed a significant and rapid reduction in anal pain, bleeding and itching compared to placebo. Furthermore, after 42 days of follow-up, the intake of painkillers was significantly lower, with a lower occurrence and persistence of oedema and thrombosis compared to the placebo group.

Physical examination of patients with EHT often reveals internal anal sphincter hypertonia, which seems to play a causal role in pain. In fact, according to the World Society of Emergency Surgery (WSES) and of the American Association for the Surgery of Trauma (AAST) guidelines on anorectal emergencies, topic muscle relaxant are suggested (21). Currently there is no evidence of benefit and no indication to the subcutaneous administration of low molecular weight heparin (22).

In this context, Perotti et al. prospectively compared the use of 1.5% topical lidocaine alone and combination of 1.5% topical lidocaine and topical 0.3% nifedipine. The evaluation on the 98 randomized patients showed in the combined nifedipine-lidocaine group a higher pain control at day 7 (86 vs. 50%, *p < 0.01*), decreased use of oral analgesia (8 vs. 54%, *p < 0.01*) and complete resolution of EHT after 14 days (92 vs. 46%, *p < 0.01*). No patient treated with nifedipine showed any systemic side effect, but only a slight local hyperemia in 4%, which disappeared with interruption of the application (23). Moreover, a prospective randomized study by Patti et al. (2) evaluated the efficacy and safety of intrasphincteric injection of botulinum toxin for pain relief in patients with EHT. The 30 randomized patients received an intrasphincteric injection of either 0.6 mL saline or 0.6 mL of a solution containing 30 units botulinum toxin. Pain intensity was significantly reduced in the botulinum group within 24 h of injection (*p < 0.001*), whereas in the placebo group a reduction was noted only from day 7. The latter group also needed a higher amount of daily analgesic tablets compared to the botulin group (2.3 vs. 1.6, *p = 0.008*). No systemic or local side-effects or anal incontinence was recorded in any patients (2).

Gallo et al. suggested the use of a polysaccharide complex, eg mesoglycan, with antithrombotic and profibrinolytic properties with the aim of reducing the post-operative thrombosis of the mucocutaneous bridges after excisional hemorrhoidectomy (24, 25). The same authors suggested the combined use of mesoglycan with local nifedipine or GTN, stool softeners, increased oral intake of fluid, oral and/or topical analgesics (12). However, further prospective studies are needed to validate its use in EHT.

## Surgical Management and Outcomes

Surgical treatment is recommended when EHT fails to respond to conservative management or, traditionally, when symptoms have been present for up to 48–72 h (10). Drainage with a radial incision and complete excision of EHT are considered the two conventional methods (3, 12), although various strategies and techniques have been attempted to reduce post-operative pain and recurrence rate.

Jongen et al. (1) retrospectively reviewed 340 patients who underwent outpatient excision of EHT under local anesthesia using a solution of mepivacaine 1%, epinephrine 0.0005% and sodium bicarbonate 8.4%. The 0.3% of these patients had post-operative bleeding controlled under local anesthesia and 2.1% developed a fistula or anal abscess. Twenty-two (6.5%) developed a recurrent EHT ≥2 months after the initial excision. After 17.5 months of follow-up, 66.4% had no anorectal complaints, 21% pruritus ani, 9.4% anal pain and 5.4% anal bleeding. Ninety-eight percent of patients were satisfied of outpatient treatment and in 79% local anesthesia was felt acceptable for another excision.

In literature two studies compared surgical and conservative managements of EHT. Greenspon et al. (15) retrospectively reviewed 231 patients with EHT, 51.5% of them were treated conservatively and the remaining 48.5% surgically (97.3% with excision and the rest with incision and evacuation of thrombus). Resolution of symptoms (pain, bleeding and perianal tumefaction) was achieved earlier by the surgical group (3.9 vs. 24 days, *p < 0.0001*). The conservative group had a higher frequency of recurrence (25.4 vs. 6.3%, *p < 0.0001*) with also a shorter time span to recurrence (7.1 vs. 25 months, *p < 0.0001*) (15). The second trial is the prospective study by Cavic et al., that randomized 150 patients into three treatments groups: topical application of 0.2% GTN, incision and excision of EHT. Comparison of postoperative pain scores revealed a less severe intensity of pain provided by classical excision, followed by topical application of GTN. However, no difference in symptomatic relief was found at 1-month follow-up between the groups, but at 1-year the percentage of patients without symptoms and recurrence was significantly higher in the excision group (26).

Two prospective randomized trials by Brown et al. and Wong et al. (27, 28) evaluated the role of stapled technique for EHT compared with conventional hemorrhoidectomy. The stapled procedure was burdened by significantly longer operation time and pain at discharge (5 vs. 1 at VAS); however, the median stay was not significantly different between the groups. In the first postoperative weeks the conventional group complained of persistent bleeding and reported significantly higher pain score, particularly on passing stool. The stapled group required a significantly shorter period to become analgesic/pain-free with a shorter time required for wound healing. This group also resumed work or activities of daily living sooner than the conventional group. At 1 year follow-up in both groups none of the patients complained of incontinence, whereas 25% of patients in the conventional group developed recurrent symptoms. The stapled group had a significantly better overall symptom improvement and were more satisfied with surgical outcomes during follow-up (**Table 1**).

**Table 1:** Main studies reporting surgical and conservative treatment in patients affected by EHT.

Study	Design	Patients No.	Mean Age	Conservative treatment	Incision	Open/closed hemorrhoidectomy	Stapled hemorrhoidopexy	Recurrence
Eberspacher	Retrospective	87	80.9	36 pts. (A): stool softeners, oral, topical analgesics, flavonoid mixture (diosmin, hesperidin)	31 pts. (B): incision	20 pts. (C): hemorrhoidectomy		12.5 months A. 19.4% B. 16.1% C. 0%
Greenspon	Retrospective	231	43.2 (A) 41.9 (B)	119 pts. (A): conservative treatment	3 pts. (B): incision	109 pts.(B): excision of the thrombosed vessel		A. 25.4% (7.1 months) B. 6.3% (25 months)
Brown	Prospective randomized	30	44–46			15 pts: hemorrhoidectomy	15 pts: stapled mucosectomy with PPH technique	
Gebbensleben	Prospective cohort study	48	43	No water, shower, bath, washcloth, wet wipes, soap, shower gel. Only smooth dry toilet paper for anal cleaning for 2 weeks.				6 months 21.3%
Perrotti	Prospective randomized	98	35	50 (study): topical 0.3% nifedipine every 12 h for 2 weeks 48 (control): topical lidocaine ointment every 12 h for 2 weeks				
Patti	Prospective randomized	30	40	15 pts. (A): Botulinum toxin injection 15 pts. (B): Placebo				12 months A. 20% B. 26%
Jongen	Retrospective	340				Excision under local anaesthesia		24 months 6.5%
Cavcic	Prospective randomized	150		50 pts.(A): 0.2% glycerlyl trinitrate ointment	50 pts. (B): Incision	50 pts. (C): Excision		12 months A. 21% B. 24% C. 5%
Wong	Prospective randomized	41	47 (A) 53 (B)			20 pts. (A): Hemorrhoidectomy	21 pts. (B): Stapled hemorrhoidectomy	12 months A. 25% B. 0%
Giannini	Prospective randomized	66	49	Mixture of diosmin, troxerutin and hesperidin (Triade H)				42 days 10%

## EHT Management in Elderly

Geriatric patients tend to be at high risk of complications because of their comorbidities (e.g., cardiovascular diseases) and frequent assumption of anticoagulants, which favor conservative management over surgical treatments. Only one study retrospectively evaluated the differences between conservative and surgical treatment (i.e., incision with evacuation of thrombosis and Milligan Morgan’s hemorrhoidectomy) for EHT in elderly (age >75). The group treated with incision reported immediate pain relief (2.6 day) followed by the excision group (7.3 days). However, the incision group was the only one that reported bleeding in 16% of patients for an average duration of 1.4 days. No recurrence was found in the excision group, with a similar recurrence between incision and conservative group (16 vs. 19% respectively) after 10 months. No major complication or anal stenosis were reported in all groups (29).

## EHT Management During the Pregnancy

EHT is one of the most important sources of anal pain during pregnancy. Around 8% of pregnant females will experience EHT, especially during the 2nd and 3rd trimesters. Risk factors are vaginal delivery, constipation, high birth weight, traumatic and /or instrumental delivery (30–32). Prevention and conservative management (fibers, stool softeners, sitz baths and topical creams) are considered the initial treatment, reserving surgical management for postpartum period. There has been reluctance in performing surgical excision, especially under general anesthesia, due to technical difficulties encountered with patient positioning as well as fear of inducing premature labor (31). Mirhaidari et al. demonstrated the safety and effectiveness of EHT excision under local anesthesia in outpatient setting. Forty pregnant females with an average gestational age of 31.7 weeks underwent excisional treatment. Twenty-one patients were complicated by a recurrence, fissure and/or hemorrhoidal tag. The recurrence rate of EHT was 32.5%, only 10% of which occurred during pregnancy. No spontaneous abortion or admission for preterm labor occurred (31).

## Conclusions

This mini review exclusively considered articles published in the last 20 years. Medical treatment has been proven effective for pain control and swelling. Different surgical treatments are recommended when EHT fails to respond to conservative management, or when symptom onset has occurred no later than 72 h from reaching medical attention. Surgical management provides rapid symptom relief, lower incidence of recurrence and longer remission interval when compared to medical treatment. Future studies and consensus-based guidelines are needed to determine the optimal treatment of EHT, particularly in special circumstances (e.g., pregnancy and elderly).
